# Bioplastics: Innovation for Green Transition

**DOI:** 10.3390/polym15030517

**Published:** 2023-01-18

**Authors:** Ana Costa, Telma Encarnação, Rafael Tavares, Tiago Todo Bom, Artur Mateus

**Affiliations:** 1 CDRSP-IPL, Centre for Rapid and Sustainable Product Development, Polytechnic Institute of Leiria, 2430-028 Marinha Grande, Portugal; 2 CQC-IMS, Department of Chemistry, University of Coimbra, 3004-535 Coimbra, Portugal; 3 PTScience, Avenida do Atlântico, N° 16, Office 5.07, Parque das Nações, 1990-019 Lisboa, Portugal; 4 Complexo Industrial VANGEST—Edifício 2, Rua de Leiria 210, 2430-527 Marinha Grande, Portugal

**Keywords:** bioplastics, biopolymers, conventional polymers, biodegradability, renewable resources, LCA

## Abstract

Bioplastics are one of the possible alternative solutions to the polymers of petrochemical origins. Bioplastics have several advantages over traditional plastics in terms of low carbon footprint, energy efficiency, biodegradability and versatility. Although they have numerous benefits and are revolutionizing many application fields, they also have several weaknesses, such as brittleness, high-water absorption, low crystallization ability and low thermal degradation temperature. These drawbacks can be a limiting factor that prevents their use in many applications. Nonetheless, reinforcements and plasticizers can be added to bioplastic production as a way to overcome such limitations. Bioplastics materials are not yet studied in depth, but it is with great optimism that their industrial use and market scenarios are increasing; such growth can be a positive driver for more research in this field. National and international investments in the bioplastics industry can also promote the green transition. International projects, such as EcoPlast and Animpol, aim to study and develop new polymeric materials made from alternative sources. One of their biggest problems is their waste management; there is no separation process yet to recycle the nonbiodegradable bioplastics, and they are considered contaminants when mixed with other polymers. Some materials use additives, and their impact on the microplastics they leave after breaking apart is subject to debate. For this reason, it is important to consider their life cycle analysis and assess their environmental viability. These are materials that can possibly be processed in various ways, including conventional processes used for petrochemical ones. Those include injection moulding and extrusion, as well as digital manufacturing. This and the possibility to use these materials in several applications is one of their greatest strengths. All these aspects will be discussed in this review.

## 1. Introduction

The use of polymeric materials is widely spread around the world. These materials have significant advantages compared with other, more conventional materials, such as metals and wood, mainly because of their properties and performance.

It is estimated that 99% of these polymeric materials come from fossil fuels. These plastics entail several issues since their primary raw material is a hazard to environment conservation [1].

The durability and degradability of these materials are two contradictory topics. For most applications, it is favourable that the material maintains specific properties throughout time, but it is also desirable to discard them easily after their use. There are some alternative processes usually used to manage this kind of waste: recycling (one of the most sustainable waste management processes but requires a controlled process to have a final product with good properties) and energy recovery (allows the production of energy by burning the waste but ends up producing toxic emissions and greenhouse gases) [2,3]. However, a massive quantity of material ends up in landfills or even abandoned, and some of it reaches the ocean. A long-term study took place on the North Atlantic Sea, where it was observed a seawater sample contained 580,000 pieces of plastic per square kilometre. This waste management has created a crisis, since landfills have a limited capacity, high costs and strict legislation [4].

The remaining percentage of plastics is produced from natural raw materials and are denominated bio-based plastics or bioplastics [1]. The use of bioplastics dates centuries ago. In 1500 BCE, Mesoamerican cultures (Maya, Aztecs) used natural rubber and latex to make containers and waterproof their clothes. However, only in 1862 was the first manmade bioplastic produced (Parkesine, a bioplastic made from cellulose), created by Alexander Parkes. The first company to produce bioplastics was Marlborough Biopolymers in 1983. They produced strips, filaments, chips, panels and powders of bacteria called Biopol. More recently, in 2018, Project Effective was launched with the goal of replacing nylon with bio-nylon, and it created the first bioplastic made from the fruit [5].

**Figure 1:**
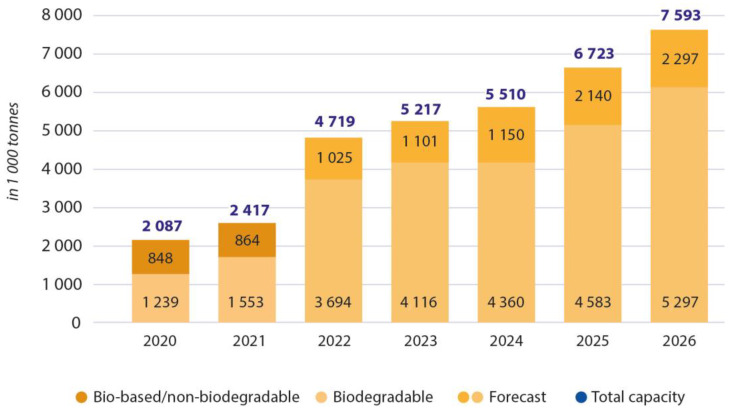
Global production capacities of bioplastics 2021–2026. Adapted from European Bioplastics, “Bioplastics Market Development Update 2021”. https://docs.european-bioplastics.org/publications/market_data/Report_Bioplastics_Market_Data_2021_short_version.pdf (accessed on 29 December 2022) [6].

Although investigations regarding bioplastics have been done for over a century, their implementation and extensive production is not yet developed. Figure 1 presents a chart of the last few years’ production and the forecast for the years to come. In 2019, 1.95 Mt of bioplastic was produced, corresponding to about 0.6% of all plastic production worldwide.

The small production of these plastics is mainly due to their more expensive manufacturing and generally inferior mechanical properties compared to fossil-based polymers. However, it is necessary to develop these materials to have a sustainable alternative to petrochemical materials [7]. This paper will discuss current scenarios and the inherent production limitations and present the pros and cons of producing and using bioplastics to replace some petrochemical-based polymers. While several reviews on biopolymers have been extensively published [8,9,10,11], the contribution of this review is to gather current knowledge in several aspects and present the latest discoveries in this topic. We have selected the most representative biopolymers and composites and present new ones. Several applications are described, and processing techniques are discussed. Moreover, some fundamental approaches to bioplastic waste management are presented. Lastly, we highlight legislation and policies that can contribute to promising future perspectives for innovation for the green transition.

## 2. Materials

**Figure 2:**
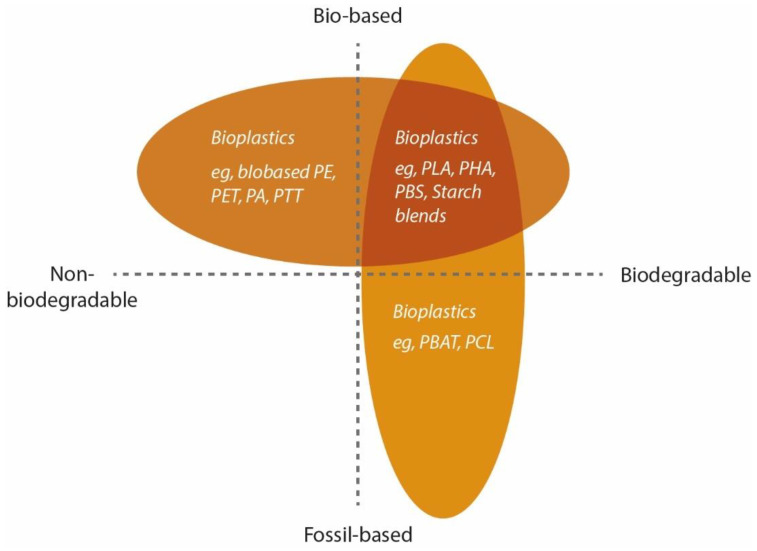
Classification of bioplastics according to The European Bioplastics Organization. Adapted from European Bioplastics, “What are bioplastics?”. https://docs.european-bioplastics.org/publications/fs/EuBP_FS_What_are_bioplastics.pdf (accessed on 29 December 2022) [13].

The European Bioplastics organization classifies bioplastics as “plastics based on renewable resources or as plastics which are biodegradable and/or compostable”. When it is possible to decompose a polymer into carbon dioxide (CO_2_), methane, water, inorganic compounds or biomass through an enzymatic process using microorganisms, the polymer is considered biodegradable. It is possible to compost some of these materials under controlled conditions [12]. Based on this definition, it is possible to organize this classification in a simple graph represented in Figure 2.

**Figure 3:**
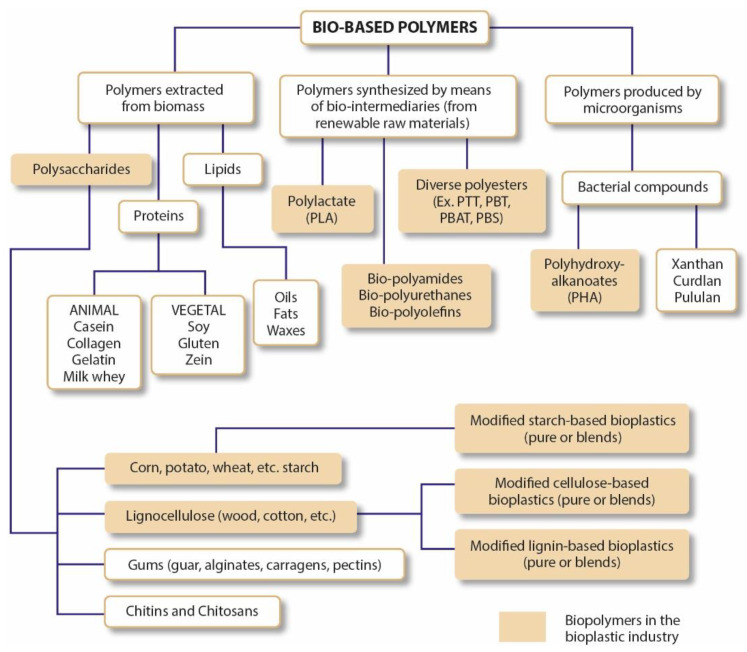
Types of bioplastics according to their raw material. Adapted from “Innovation and industrial trends in bioplastics”, Polymer Reviews vol. 49, no. 2, pp. 65–78, April 2009 [14].

In the bioplastic group, they can be classified under three different classes, as shown in Figure 2: (1) polymers originated from biomass materials, and they can be either modified or not; (2) polymers extracted from natural or genetically modified microorganism production and (3) and polymers produced from renewable raw materials with the involvement of bio-intermediaries. Although only some of these materials are available on a commercial scale, the most used bioplastics are based on cellulosic esters, starch, polyhydroxy butyrate (PHB), polylactic acid (PLA) and polycaprolactone (PCL). Figure 3 shows some of the most used bio-based polymers according to their base of production and raw material [14,15,16].

Some biopolymers have properties comparable with conventional plastics, such as LDPE (low-density polyethylene), PS (polystyrene) and PET (polyethylene terephthalate); some of these properties are important to predict the behaviour of the material during use and the proper conditions to process it. Some of these characteristics, listed in Table 1, are the glass transition temperature (Tg), melting temperature (Tm), tensile strength, tensile modulus and elongation break [2].

Another characteristic important to consider is the rate of crystallinity of the polymer. It influences a vast quantity of essential properties such as hardness, modulus, tensile strength, stiffness, crease point and the melting point, making it important to pay special attention to this property [2].

The values in Table 1 are generic, since it is possible to obtain materials with different properties with different combinations of monomers or through chemical derivatization or introduction of additives such as plasticizers, stabilizers, fillers, processing aid and colourants.

Table 2 summarizes some characteristics that may be important for specific applications listed as guidelines. Some typical applications and the degradability of the discussed bioplastics are also included in Table 2 [27].

### 2.1. Starch

**Figure 4:**
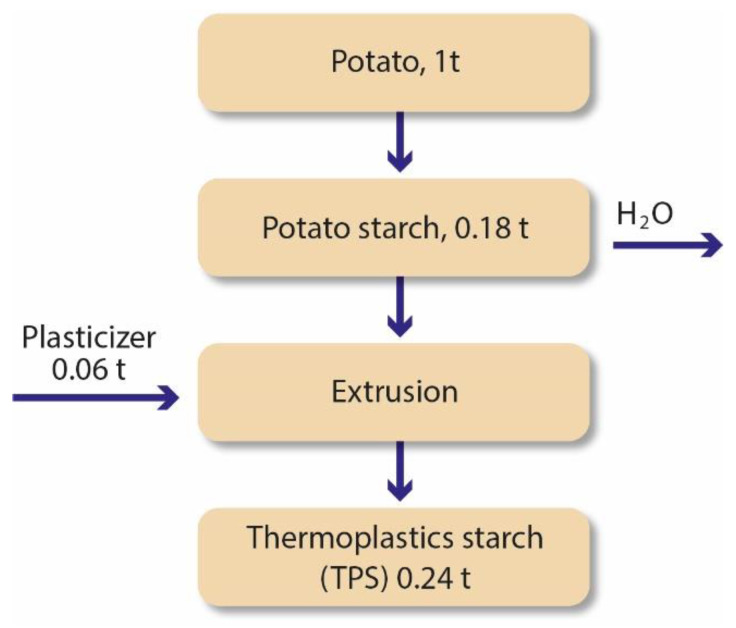
Production flow chart of thermoplastic starch (TPS). Adapted from “Bioplastics: Development, Possibilities and Difficulties”, Environmental Research, Engineering and Management, vol. 68, no. 2, July 2014 [36].

The synthesis of this bioplastic began in the 1970s and is now produced worldwide by companies such as Futerro, Novamont, Biome and Biotec [26,27]. Starch is one of the common names for carbohydrates, along with sugars, saccharides and polysaccharides. They are formed by photosynthesis when CO_2_ reacts with water. The chemical symbol is generically represented as Cx(H_2_O)y, where x and y are numbers between 3 and 12. Starch is a type of polysaccharide obtained from floral sources [28]. The production of polymeric film from starch requires a significant quantity of water or plasticizers (glycerol, sorbitol). They are used widely worldwide as a substitute for PS in several thermal and mechanical applications. There are a lot of different possible sources of starch, but the main ones used are corn, wheat, cassava and potatoes, with 82%, 8%, 5% and 5% starch, respectively. The raw material is prevalent, and the production process allows obtaining large quantities of a biodegradable thermoplastic-like material (TPS) with a fair ease of management. Although starch is not a thermoplastic, starch-based bioplastics melt at high temperatures (91–180°C) and tend to be fluid under shearing. The use of plasticizers helps to achieve this behaviour, making it possible to process the material with injection moulding, extrusion and blow moulding. Plasticizers work embedded between polymer chains, which soften the material and lowers the glass transition temperature by spacing the polymer chains apart. There are several processes involved in the conversion of starch into thermoplastic, such as gelatinization, melting, water diffusion, granule expansion, decomposition and crystallization. The thermoplastic material forms in the presence of heat and shearing forces. The energy absorbed melts the original structure and creates new bonds between the starch and the plasticizer. When the mixture cools down to room temperature and the granules reswell, a new granule structure is formed, and the thermoplastic material is produced. The final material has both amorphous and crystalline regions. Cereplast is a producer of TPS that collects starch from tapioca, corn, wheat and potatoes. Out of 1 t of potatoes, they are capable of gathering 0.18 t of starch, which produces 0.24 t of TPS when adding 0.06 t of plasticizer, a process summarized in Figure 4 [12,30,31,32,33,34,35].

Since water has a plasticizer effect on starch, one of the problems of the use of starch is the effect water and humidity have on it, creating, for example, variations in its mechanical properties and low resistance to impact. Some derivates of starch have high permeability to moisture and degrade rapidly in specific applications; solutions used to avoid these problems might make the final material expensive. Throughout time, the material’s properties change even when temperature and moisture are controlled, lowering the elongation break and increasing the rigidness [30].

Examples of products made of starch bioplastic are grocery bags and trays (rigid or foamed) used to pack fruits and vegetables. One important material widely used is paper foam, used to pack items where product protection is essential, for example, in egg boxes and the packaging of electronic devices [37,38].

### 2.2. Cellulose

This polymer is a biodegradable polysaccharide made from wood pulp or cotton linters. Cellophane film starts by dissolving the raw material in a mixture of sodium hydroxide and carbon disulphide and recast the obtained product into an acid solution (sulfuric acid) [12,32].

Pure cellulose bioplastic is very hard to be produced; it is not possible to make it in an industrial environment with standard processes such as thermoforming or dissolution due to its strong and highly structured intermolecular hydrogen bonding network. For this reason, it is usually produced industrially as cellulose derivatives, such as cellulose esters or ethers, which requires extra time and costly chemical purification steps [39]. Some of these derivates are cellulose nanocrystals (CNC), nano-fibre cellulose (NFC), cellulose acetate butyrate, cellulose acetate and bio-PE, and they are produced by the esterification or etherification of hydroxyl groups. A lot of derivates need additives to produce thermoplastics, and some of them are water-soluble [12,40].

Cellophane is transparent, and it can be pigmented. It is common to use it as candy wrappings, laminates, flower wrapping and pack products ranging from cheese to coffee and chocolate [33].

### 2.3. Polyhydroxyalkanoates (PHA)

PHA is a family of biodegradable thermoplastic polymers where more than 160 different monomeric units were identified. The most common one is polyhydroxybutyrate (PHB). PHA is a material regularly used to replace conventional polymers due to their similar chemical and physical properties. This biopolymer is produced by the fermentation process in microbial cells (such as *Cupriavidus necator*, *Bacillus* sp., *Alcaligenes* sp., *Pseudomonas* spp., *Aeromonas hydrophila*, *Rhodopseudomonas palustris*, *Escherichia coli*, *Burkholderia sacchari* and *Halomonas boliviensis*), and the polymeric material is then recovered using solvents (chloroform, methylene chloride or propylene chloride) [8,33]. Corn, whey, wheat and rice bran, starch and starchy wastewaters, effluents from olive and palm oil mills, activated sludge and swine waste are some examples of material sources for the fermentation process. Although the conditions of the fermentation process depend on the demands of the microbes, temperatures of 30 °C to 37 °C, along with low stirrer speeds (resulting in low dissolved oxygen tension), are the conditions used.

**Figure 5:**
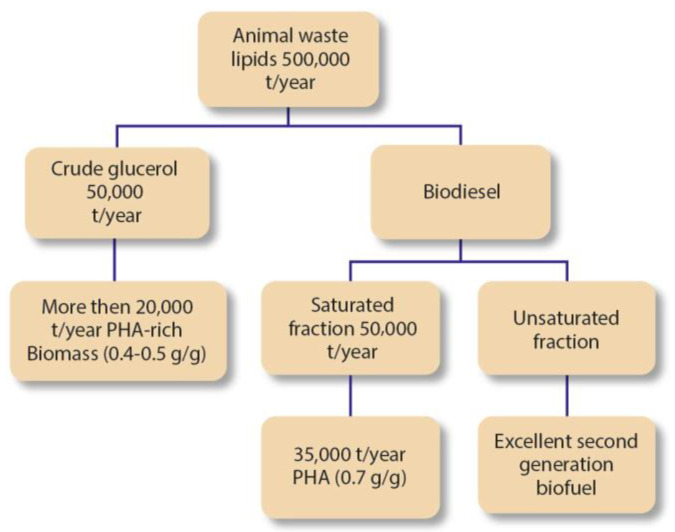
Production flow chart of PHA from waste streams from slaughterhouses. Adapted from “Bioplastics: Development, Possibilities and Difficulties”, Environmental Research, Engineering and Management, vol. 68, no. 2, July 2014 [32].

Several national and international programmes foster the development and advancement for a green transition. That can be expressed in several projects’ results. In 2010, the “Animpol” project was developed by the European Commission with the goal of developing an efficient process that could convert waste streams from slaughterhouses into improved biodiesel and biodegradable high-value polymeric materials, such as PHA. The Consortium was able to produce 35,000 t per year of PHA from 500,000 yearly t of animal waste on an industrial level, as represented in Figure 5. The introduction of this process in the bioplastic production industry would mean that some of the solvents used in the production of bioplastics would be eliminated, and the slaughterhouse waste could be useful to produce added-value products, while nowadays, this waste material is simply burned [8,32,33,38].

PHB is produced by bacteria, algae and genetically modified plants through enzymatic processes. The process starts with the condensation of two molecules of acetyl-CoA into acetoacetyl-CoA, which is then reduced by acetoacetyl-CoA reductase to produce β-hydroxybutyryl-CoA. PHB is obtained by the polymerization of β-hydroxybutyryl-CoA. To harvest the PHB, it is necessary to destroy the cell, since it is present as cysts within the cytoplasm of the cell [33,39,40].

Due to its characteristics, PHA is extensively used in medical fields, especially in tissue engineering. For example, PHA is used in long-term dosage of drugs, medicines, hormones, insecticides and herbicides, as osteosynthetic materials in the stimulation of bone growth owing to their piezoelectric properties in bone plates, surgical sutures and blood vessel replacements. Other products made of this biopolymer are composting bags, food packaging, diapers and fishing nets [33]. Tianan Biopolymer, BASF, Tepha and Biocycle are some examples of companies that produce this type of bioplastics [41].

### 2.4. Polylactide (PLA)

PLA was initially used in combination with polyglycolic acid (PGA) under the name Vicryl during the 1970s, but its discovery goes back to 1932 when it was discovered by Carother. At the time, he produced a low molecular weight polylactide by heating lactic acid under vacuum while removing the condensed water. Nowadays, most of the PLA produced is used in packaging (about 70%), although its application in other fields has been increasing, especially in fibres and fabrics. The leading producer of PLA in the world is NatureWorks^®^ (USA), but other companies stand out, such as Ingeo, Toyobo, Dai Nippon Printing Co., Mitsui Chemicals, Shimadzu, NEC, Toyota, Biofront (Japan), PURAC Biomaterials, Hycail, Biofoam (The Netherlands), Galactic (Belgium), Cereplast, FkuR, Biomer, Stanelco, Inventa-Fischer (Germany) and Snamprogetti, Hisun (China) [2,30,42].

Similar to PHA, PLA is one of the bioplastics considered to have significant potential to be widely used as a replacement to several fossil fuel-based polymers, such as LDPE and high-density polyethylene (HDPE), PS and PET [8].

PLA involves different types of sciences to be produced: agriculture, for the growth of the crops; biological, during the fermentation process; and chemical, for polymerization. The PLA monomer is called lactic acid (2-hydroxy propionic acid), and it has two configurations, L(+) and D(−) stereoisomers, produced by the bacterial fermentation of carbohydrates (homofermentative and heterofermentative). Lactic acid is produced using one of two processes, fermentation or chemical synthesis. The first one is usually used industrially, since it does not depend on other processes’ by-products, it produces L-lactic acid stereoisomer easier and the manufacturing costs are not as high as the synthesis process. The homofermentative method is also preferable, since it creates less by-products and lactic acid with greater yields, and pure L-lactic acid is used to produce PLA. The production procedure uses *Lactobacillus* genera such as *L. delbrueckii*, *L. amylophilus*, *L. bulgaricus* and *L. leichmanii* under specific conditions (a pH range of 5.4 to 6.4, a temperature range of 38 to 42 °C and a low oxygen concentration).

**Figure 6:**
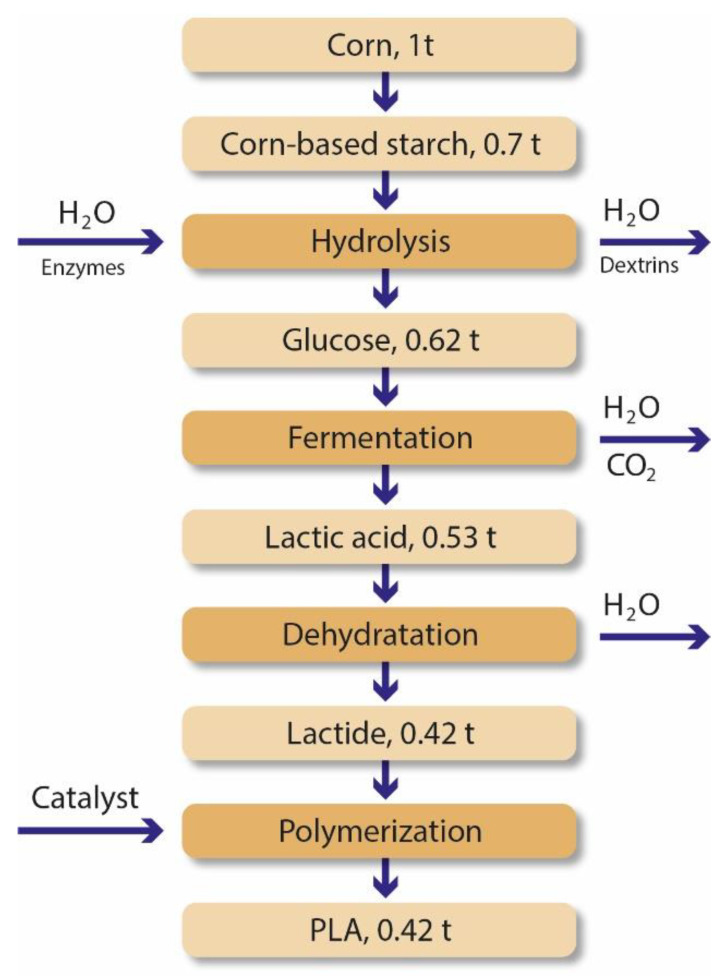
Production flow chart of PLA by synthesizing corn-based starch. Adapted from “Bioplastics: Development, Possibilities and Difficulties”, Environmental Research, Engineering and Management, vol. 68, no. 2, July 2014 [32].

After the production of lactic acid, it is then polymerized into PLA. There are three possible processes to do the polymerization: direct condensation polymerization, direct polycondensation in an azeotropic solution and polymerization through lactide formation. High molecular weight PLA with good mechanical properties is not easily achieved using the direct condensation polymerization method. It involves the esterification of lactic acid with some solvents under progressive vacuum and high temperatures, where water is removed. The second process is a more feasible way to produce PLA with high molecular weight. The azeotropic solution reduces the distillation pressure, and using molecular sieves helps the separation of the PLA and the solvent. Lastly, polymerization through lactide formation is also used in the industrial environment to produce high-weight PLA. Lactide is a cyclic dimer formed by removing water under mild conditions and without solvent. As shown in Figure 6, it is possible to produce 0.42 tons of PLA from 1 ton of corn using processes such as hydrolysis, fermentation, dehydration and polymerization [32,33,43,44,45].

PLA is widely used in the food packaging industry for short and long shelf-life products. This bioplastic and its blends are also used to make implants, plates, nails and screws for medical surgery. The application fields are expanding to textile, cosmetic, automobile industries and the household [33].

### 2.5. Polycaprolactone (PCL)

PCL is a synthetic polyester that is produced from crude oil. Since it is a biodegradable polymer, it is considered a biopolymer.

PCL is regularly used in tissue engineering and biomedical applications due to its blend compatibility, absorbability, good solubility and low melting point. This is a hydrophobic and semi-crystalline material; its crystallinity depends on its molecular weight (high molecular weight means low crystallinity), which is possible to control using low molecular weight alcohols. This biopolymer is characterized by its ease to manufacture and shaping, tailorable degradation kinetics and mechanical properties, making PCL distinguish itself from other bioplastics.

Initially, when it was studied in the 1930s, this polymer was relatively popular, but soon, its use decreased significantly for a long time due to its weak mechanical properties in comparison to other resorbable polymers such as polylactides and polyglycolide. Recently, this material has regained great interest because of its application in the field of tissue engineering widely developed in the 1990s. It not only possesses superior rheological and viscoelastic properties when compared to other polymers, but its ease of processing also stands out.

Although PCL is a bioplastic biodegraded by specific bacteria and fungi present in outdoor environments, this polymer is not degradable in animal or human bodies due to the lack of those organisms. This particularity makes PCL an excellent material to be used in medicine and tissue engineering fields of study; its degradability is relatively slow when compared to PLA, PGA and other resorbable polymers, making it an excellent material to be used “for long-term degradation applications delivery of encapsulated molecules extending over a period of more than 1 year”. The rate at which this drug release is achieved may be manipulated by combining PLC with cellulose propionate, cellulose acetate butyrate, PLA or polylactic acid-co-glycolic acid [46].

### 2.6. Protein-Based

Protein is a heteropolymer of amino acids with a fibrous and globular structure arranged by hydrogen, covalent and ionic bonds. Protein-based materials have great mechanical and barrier to gas and aroma properties than lipids and polysaccharides. By incorporating keratin, the material produced has thermal stability, mechanical properties and flame resistance. Due to their abundance, biodegradability, nutritional value and better film development capability, packaging is one of this material’s main applications [47]. Other applications include matrices for enzyme immobilization or controlled-release devices and in fields where water absorbency and retention are important, such as water-absorbent materials in healthcare, agriculture and horticulture. With further technological development, packaging technology, natural fibre reinforcements, nanotechnology and innovative product design could be fields to be developed using this type of bioplastic.

To produce products with this material, there are two possible processes, the casting method (or physicochemical method) and the mechanical method (or thermoplastic processing). The first process divides itself into three different steps, starting with using chemical or physical rupturing agents to break the intermolecular bonds that stabilize polymers in their native forms; next, the mobile polymer chains are rearranged and oriented to the intended shape. Finally, the three-dimensional network is stabilized by allowing the formation of new intermolecular bonds and interactions. The second method involves mixing proteins and plasticizers to obtain a dough-like material [31,47]. Techniques that can be used to observe the molecular reorganization and orientation are based on time-resolved small-angle X-ray scattering (SAXS) that allows analysing of the anisotropy. Usually, these techniques are available in research facilities that allow in situ observation. It is possible to, through in situ experiments, observe the molecular rearrangement and reorganization that impose levels of preferential orientations [48]. The time-resolved SAXS allows monitoring the evolution (in time) of changes in the morphological organization.

### 2.7. Polyamide 11 (PA 11)

PA 11 or nylon 11 is a nonbiodegradable bioplastic produced from renewable material, such as castor oil. The polymer is obtained by the polymerization of 11-aminoundecanoic acid. Although it is nonbiodegradable, PA11 and its composites can be recycled. Due to its nonbiodegradability, the PA11 has greater longevity than most bioplastics. This characteristic, along with the high melting point (200 °C) and mechanical and chemical stability, makes it possible to apply it as reinforcement material in the manufacturing of natural gas piping, water tubing, electrical cables, clips and wires in aerospace and automobile industries, metal coatings, footwear, badminton racket strings and shuttlecocks. Other important characteristics are its good resistance to oil and water, high resistance to ionization radiation, strong resistance to different chemicals, fuels and salt solutions and its resistance to abrasion and cracking. It has low heat resistance and rigidity, low resistance to ultraviolet radiation and weak resistance to acetic acid and phenols, and its electrical properties are highly dependent on the moisture content. However, the market price is relatively higher than other polyamides. One of the main PA11 producers is Arkema [47,49,50].

### 2.8. Spidroin—Spider Silk

Spiders can, in a fraction of a second, under ambient conditions and from renewable resources, create a material which mechanical properties outperform any manufactured material: spider silk. It has the potential to be used in a wide variety of fields, for example, for making high-performance textiles and sports goods, durable components for robotics, ropes and reinforcements of composite materials and for applications in medicine (spider silk enhances wound healing and has successfully been used to bridge critical size nerve defects and as fascia replacements in animal models) [39,51,52]. However, it is currently not possible to farm spider silk efficiently on large scales because of spiders’ cannibalistic nature, the difficulty of breeding, the low production rate in captivity and the collection of silk from spider webs is very time-consuming and not efficient enough for production. Genetic engineering is the most promising way to produce this material. The plan is to express the spider silk protein (spidroin) into different kinds of hosts, such as bacteria, yeast, plant and in the milk of transgenic mammals [40,51,52]. This technique was widely researched using *Escherichia coli*, a well-established host for the industrial-scale production of proteins. Yet, these materials produced do not have as good mechanical properties as spider silk, so new methods need to be developed. Although spider silk always looks the same to the naked eye, there are several different types of thread; the same spider can produce up to seven different types of silk, each one with different mechanical properties. The strength of these natural threads ranges from 0.02 to 1.7 GPa, and its extensibility varies between 10 and 500%. Additionally, using the same natural fibres, those spun technically show different properties than the natural fibres created by the spider, which shows that this is an important process and is done differently than the technology usually used. Despite a biomimetic spinning process to process the silk not existing, there are other alternatives to use this material since recombinant spider silks can self-assemble into non-natural shapes such as spheres, capsules, films, non-wovens or hydrogels. There are other concerns regarding solubility, storage and assembly of the underlying spider silk proteins [53].

## 3. Bioplastics Composites

The synthetic assembly of two or more materials, a matrix binder and selected reinforcing agents, is used for various applications. The goal is to overcome the weaknesses and increase versatility. The most common fibres exercised in recent times are glass fibres, carbon fibres, aramid fibres, natural fibres, nylon and polyester fibres. There is a clear advantage to using natural-based fibres; for example, they are biodegradable, renewable, available in bulk, cheaper and lighter [54,55,56].

### 3.1. Coating

Coating bioplastics is an excellent technique to improve some of the properties of these materials; it is specially used to enhance the barrier properties. By applying a thin layer of other polymers on top of the bioplastic, the tensile strength and elasticity can be improved, as well as increasing oxygen and water vapour permeability and resistance. Some examples of usually used coatings are listed below:

- PLA barrier properties (oxygen and water vapour) can be improved by applying a PLA-Si/SiOx, AlOx (aluminium oxide), PCL-Si/SiOx or PEO-Si/SiOx (polyethylene oxide) coating.
- When coated with PLA, SPI (soy protein isolate) films tensile strength increases from 2.8 to 17.4 MPa, and the elongation went from 165.7% to 203.4%. However, the water vapor permeability decreases 20- to 60-fold, depending on the PLA concentration in the coating solution.
- Nitrocellulose or PVdC (polyvinylidene chloride) coating on cellophane is also used to improve oxygen and water vapour barrier properties.
- Coating acetylated cellulose film with PHB increases elastic modulus and tensile strength for films containing 10% or more PHB and a better strain at break for films containing 15% or more PHB while lowering the water vapour permeability values [8].

### 3.2. Nanocomposites

**Figure 7:**
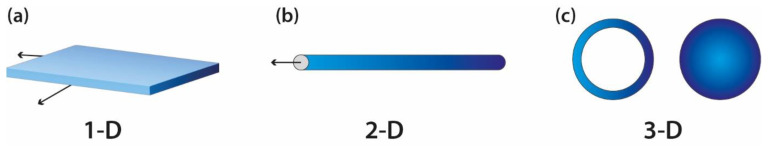
Nanoparticle geometries: (**a**) layered particles (1D), (**b**) acicular or fibrous ones (2D) and (**c**) isodimensional nanoparticles (3D). Adapted from “Block copolymer nanocomposites” [57].

For a composite to be considered a nanocomposite, it must have at least one of its types of particles with dimensions in the nano range. The composite can be classified as polymer layered crystal nanocomposites (Figure 7a), nanotubes or whiskers (Figure 7b) and isodimensional nanoparticles (Figure 7c), according to the number of dimensions it has: three, two or one, respectively [2].

The use of these materials as reinforcement to other polymers depends on the capacity of the matrix (continuous phase) to interact with the fibre (discontinuous phase). There are several ways to mix the phases of the composite; one of them is situ polymerization, which involves the dissolution of the nanoparticles in the monomer solution before polymerization; the addition of the nanoparticles during the extrusion process, a process called melt intercalation; or solvent intercalation (use of a solvent to enhance the affinity between the nanoparticles and the matrix). These processes help change some of the composite properties according to the intention.

**Figure 8:**
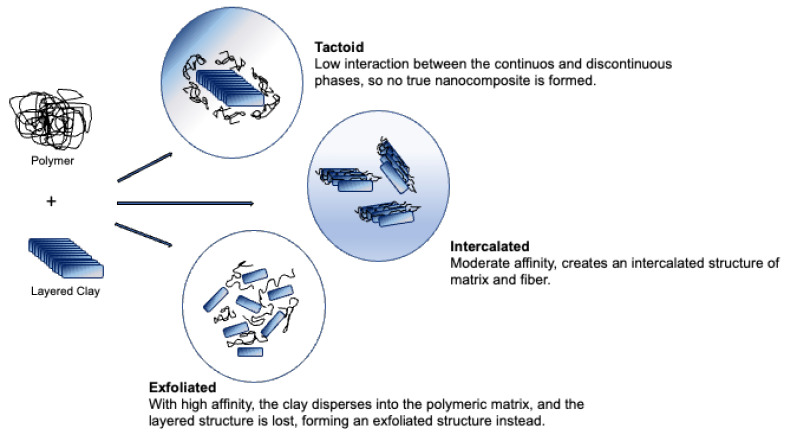
Structures of polymer nanoclay composite.

Nanoclays are one of the most used fibres as reinforcement of bioplastics. They are usually used as layered particles of 1 µm. Different affinities between the matrix and the nanofibers create different interactions: tactoid, intercalated and exfoliated. The first case occurs when the interaction between the continuous and discontinuous phases is low; this happens because the clay interlayer does not expand within the matrix, so no true nanocomposite is formed. When the affinity is moderate, it is possible for a part of the polymer to penetrate the clay interlayer, since there was some level of expansion of the fibre; this creates an intercalated structure of matrix and fibre. The last situation entails a high affinity, the clay disperses into the polymeric matrix, and the layered structure is lost, forming an exfoliated structure instead [8] (Figure 8).

**Figure 9:**
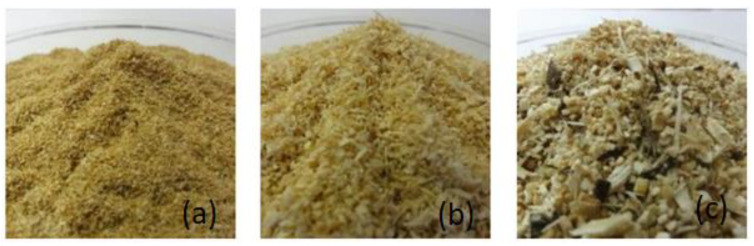
Different batches of sawdust used (**a**) (less than 0.7 mm), (**b**) (between 0.7 and 1.4 mm) and (**c**) (between 1.4 and 2.8 mm). From the project EcoPlast.

It is also possible to create composites using bigger particles as reinforcement. The project EcoPlast works with melt intercalation using natural fibres (sawdust, cellulose and cork) and biodegradable polymers as matrices (such as PLA, PBS (polybutylene succinate), starch, cellulose and PCL) in several different combinations. The materials used are represented in Figure 9. They are within different ranges of dimensions and densities, where (a) (less than 0.7 mm) and (b) (between 0.7 and 1.4 mm) are quite homogeneous. At the same time, (c) (between 1.4 and 2.8 mm) has very different particles in shape, size and colouring.

The dispersion of the fibres is affected by the hydrophobic/hydrophilic character of the polymer and the clay; this can be adapted with chemical modifications such as cationic exchange, ionomers, block copolymers adsorption and organosilane grafting. Since a high surface-to-volume ratio leads to better polymer properties, the exfoliated structure is preferred.

Some of the properties affected are the elongation at break (especially when using PLA film as matrix); barrier properties, explained by the confinement effect (the molecules of the polymeric matrix penetrate the dispersed nanoparticles, creating a denser material and creating a more tortuous path for the water and gas molecules to travel through) and thermal stability [8].

### 3.3. Cellulose

Adding cellulose to the bioplastic is another way to influence some properties of the final material. It is possible to have good adhesion between the fibre and the matrix due to the chemical similarity between starch and natural fibres. The main effect this addition has on the composite is the reduction of water vapour permeability due to the fibres’ highly crystalline and hydrophobic character, also affecting the Young’s modulus, tensile strength and the elongation break [8].

## 4. Processing

To achieve the maximum possible benefits of bioplastics, the processing of these materials is well established for each one of them, differing between them according to the characteristics of the specific bioplastic [2].

When processing bioplastics, the technologies used are the same as conventional polymers; it is only necessary to adapt the parameters used for the specific material intended to use [58]. Depending on the material, some processes might not be efficient, sustainable or economically reasonable. That is why it is essential to further research the processability of biopolymers to make their range of applications wider [59]. Table 3 summarizes some of the most common processes where bioplastics might be modified.

### 4.1. Injection Moulding and Extrusion

The possibility of processing bioplastics via injection moulding or extrusion is not very different compared to processing conventional polymers. Each type of bioplastic has its own chemical structure, so each material will have different parameters, the same as happens with conventional plastics. Only a few conditions require some attention; for example, some bioplastics are sensitive to moisture or heat exposure; therefore, it is essential to control the drying process of the bioplastics and the long-time cycles in the case of injection moulding. Thus, it is important to consider these characteristics when choosing materials, equipment, and resources [60].

### 4.2. Digital Manufacturing

**Figure 10:**
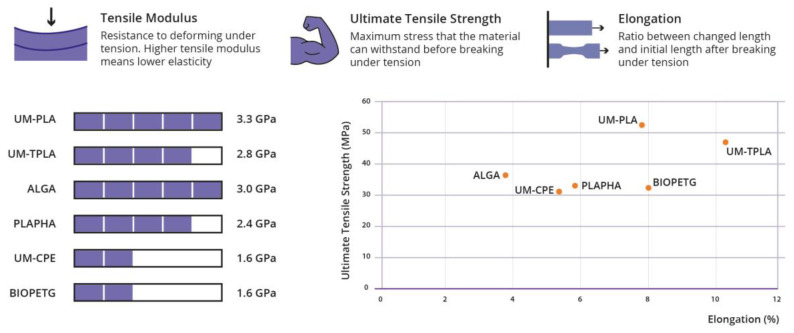
Material Guide for Green 3D printing: mechanical properties. Adapted from “3D Printing with Bioplastics”, 2020 [61]. Mechanical properties for materials PLAYPHAB and QMNI are estimated to be similar to PLAPHA and UM-TPLA respectively.

**Figure 11:**
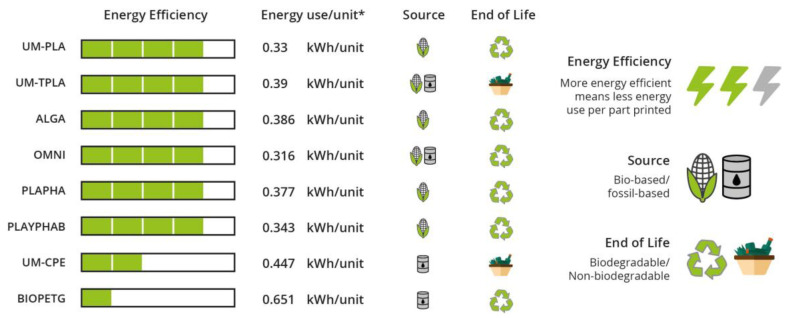
Material Guide for Green 3D printing: energy consumption and some material characteristics. Adapted from “3D Printing with Bioplastics”, 2020 [61]. * One unit refers to this reference part used for universal comparisons.

**Figure 12:**
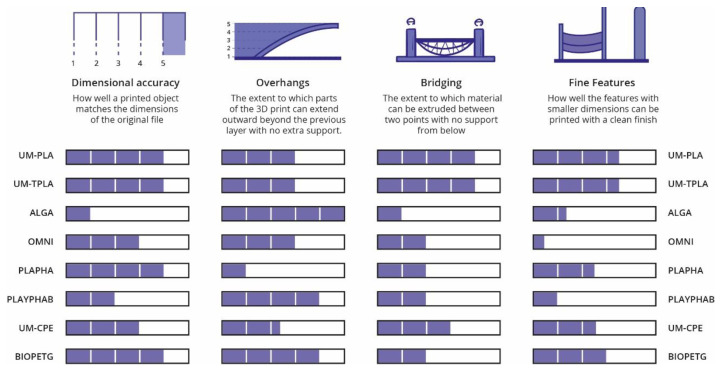
Material Guide for Green 3D printing: print quality. Adapted from “3D Printing with Bioplastics”, 2020 [61].

Several research studies have been developed in the past years with the goal of evaluating the possibility of integrating new bioplastics into production processes usually used to transform conventional polymers. One in specific was conducted by Sneha Gokhale [61]. The objectives were to “Understanding sustainable 3DP (3D printing) in the context of bioplastic filaments; Testing commercial bioplastic filaments for sustainability and material properties; Guiding users in the industry towards green 3DP material and process choices”. To achieve this, several polymeric materials were tested in three different phases, evaluating their energy consumption when processed by FDM (Fused Deposition Modelling); comparing their printability and dimensional accuracy and rating their mechanical properties (tensile modulus, ultimate tensile strength and elongation). The materials chosen to test were three Ultimaker standard materials (from the FDM machine used): UM-PLA, UM-TPLA (talc-injected PLA) and UM-CPE (chlorinated polyethylene), and five materials available on the online market: ALGA (PLA + Algae), OMNI (PLA based blend), PLAyPHAb (PLA + PHA from 3DPrintLife), PLAPHA (PLA + PHA from Colorfabb) and BioPETG (bio-polyethylene terephthalate glycol). The obtained results are summarized in Figure 10 (about mechanical properties), Figure 11 (print quality) and Figure 12 (regarding the energy consumption and some material characteristics) [61].

PLA-based materials bought scored similarly to the standard Ultimaker PLA for print quality and tensile properties. Although the print quality is likely to improve when building simpler parts. While BIOPETG, the material used to compare with UM-CPE behaved slightly better. During the tests, it was verified that the heating of the build plate of the printer was the parameter that consumed the most energy. With this information, it was possible to say that materials that require lower building plate temperatures consume less energy, making them more eco-friendly. It was possible to conclude that new biomaterials have characteristics similar to conventional materials, making it possible to substitute these with greener materials [62]. This technique is widely used to produce a variety of biomedical devices, such as orthopaedic implants, thanks to the possibility to build manufacturing customized, low-volume and complex implants. Some filaments are already made from biological sources and may utilize waste material from producing beer or coffee grounds as filling. This makes the process eco-friendlier [63].

### 4.3. Electrospinning

Electrospinning is a relatively inexpensive process used since the 1930s to produce fibres on the micro and nano scales using a high voltage (20 kV) as an electrostatic field on a polymer solution. The polymeric solution becomes electrified and stretches, becoming a thin fibre. Although it has been under development for a long time, it is still in its relative developmental infancy in industrial application.

An electrospun mat is usually made with a carrying polymer, ensuring that the mat is stable and able to incorporate other components. Bioplastics are widely used in biomedical applications since some materials are required to be biodegradable or biocompatible; PLA is one of the most commonly used biopolymers [46,64].

## 5. Applications

**Figure 13:**
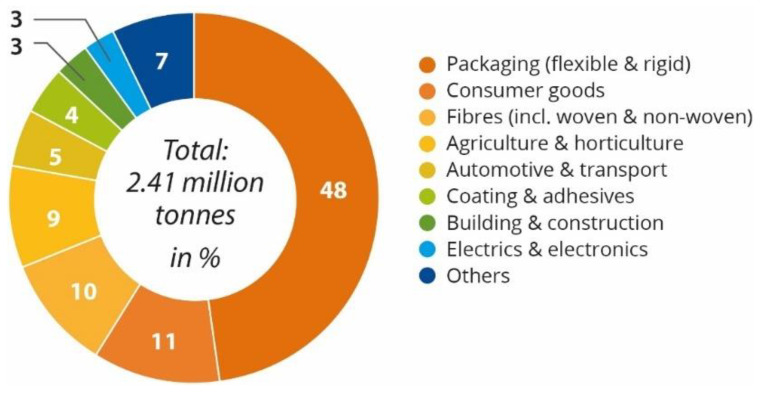
Global production capacities of bioplastics in 2021 (by market segment). Adapted from European Bioplastics “Global production capacities of bioplastics in 2021 (by market segment)”. https://www.european-bioplastics.org/wp-content/uploads/2021/11/Global_Prod_Market_Segment_circle_2021.jpg (accessed on 29 December 2022) [65].

The applications of bioplastics are several (Figure 13), for example, food packaging (with 48% or 1.15 Mt of the total bioplastics market in 2021), consumer goods (11%), fibres (10%), agriculture (9%), automotive (5%), coating and adhesives (4%), construction (3%), electronics (3%) and other sectors (7%). It is expected that bioplastics will grow and the fields of application expand [6].

### 5.1. Medical Industry

Bioplastics are an excellent replacement for conventional polymer, as they may cause less allergies than the chemical-based products usually used. They are a sustainable material for the large amounts of one-time-use products used. Since some bioplastics may be breathable and allow water vapour to permeate and be waterproof at the same time; they can be used as sanitary products such as diaper foils, bed underlay and disposable gloves [33].

### 5.2. Food Packaging Industry

The use of bioplastics in the food packaging industry has been increasing rapidly. The materials used in these types of applications require some specific characteristics to achieve the product’s intended shelf-life time and respect the food safety regulations. The mainly used bioplastics are PLA, starch and cellulose-based [33].

### 5.3. Agriculture

Some crops require using blankets of biodegradable plastic on part of their fields to increase their product yield. Usually, petroleum-based polymers are used, but bioplastics can achieve the same objective and do not leave residues, unlike conventional polymers. This makes it possible to reduce labour and disposal costs [33].

## 6. Bioplastic Waste Management

The waste management process of bioplastics is not as simple as it may seem. Although some of the process’s parameters are similar to the ones used for conventional plastics, it is not possible to mix these two types of materials without contaminating either the material itself or the environment. Another big misconception is the idea that there is no harm in dumping bioplastics anywhere with degradation assured. This only applies to some biopolymers, such as those made from seaweed.

**Figure 14:**
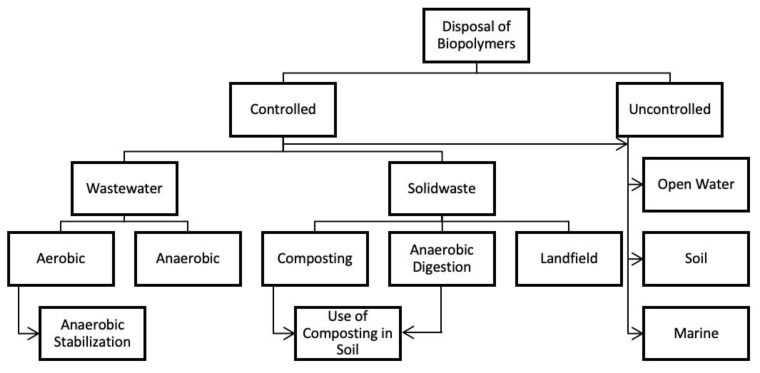
Waste management process of biopolymers. Adapted from “Bioplastics: A boon or bane?” Renewable and Sustainable Energy Reviews, vol. 147. Elsevier Ltd., 1 September 2021 [31].

The proper way to treat bioplastics is to recycle them mechanically, chemically, or organically, depending on the material’s capacity to compost or biodegrade (Figure 14). If a material is compostable, it is possible to obtain enriched compost (a valuable material) under industrial composting conditions. If the material is not compostable, the ideal process to use is chemical or mechanical recycling [31,66,67].

### 6.1. Mechanical and Chemical Recycling

Physical or mechanical recycling is already an established technology. The main problem with the mechanical recycling of bioplastics is that it is not possible to obtain a good quality material if more than one type of material is mixed; for example, PLA is a material widely used in packaging, and it is possible to recycle it mechanically; however it is difficult to distinguish it from materials such as PET by mere appearance, and this means that it would be necessary to add additional labelling to enable a correct separation by the consumers, but before that, it is needed to create a separate PLA recycling stream.

In chemical recycling, unlike the mechanical process, the aim is to reuse carbon and biogenic substances obtained from waste material to synthesize new plastic materials throw treatments that can involve hydrolysis/solvolysis, hydrothermal depolymerization and enzymatic depolymerization. Some of the advantages of chemical recycling are related to the simplicity of the overall process, and it does not require sorting or thermomechanical degradation, and it is not a process sensitive to material impurities [31,67].

### 6.2. Composting

Some materials are specifically designed to be compostable or organically recyclable; the final product is an enhancer to the soil, providing nitrogen, potassium, phosphorus and organic matter to the soil. This is an aerobic process divided into three stages, mesophilic, thermophilic and maturation. The application of these materials in single-use objects, such as bags, food packaging and cutlery strengthens their industrial utilization. Mechanical processing is necessary to separate missorted materials, reduce particle sizes for better bioavailability and mix different organic substrates for optimal dry matter content and C/N-ratio. It is possible to obtain high-value products that can be used as a soil amendment (due to the high capacity to hold water because of its organic matter content), biogas, hydrogen, ethanol and biodiesel.

The composting process at home is very difficult to control, which may result in the formation of methane gas. This process is more variable and less optimized than industrial composting, and the temperature achieved is rarely more than a few degrees Celsius above the ambient temperature [66,67].

### 6.3. Anaerobic Digestion

Anaerobic digestion aims to degrade organic wastes to biogas and digestate through four successive phases: hydrolysis, acidogenesis, acetogenesis and methanogenesis. The process can be operated at psychrophilic (18–20 °C), mesophilic (35–40 °C) and thermophilic (50–60 °C) temperature regimes, although the last two conditions are more efficient for the degradation of the bioplastics [67].

### 6.4. Waste-to-Energy (WTE)

The waste-to-energy process, also known as incineration, is highly influenced by the capacity of the polymer to degrade in terms of its sustainability, and 56% of the energy comes from the incineration of biogenic organic MSW (municipal solid waste), which means that at least half of the process products does not contribute to an increase of CO_2_ in the biosphere; in other words, “incineration of bio-based waste emits CO_2_ which was recently captured and will be captured again when new bio-based products are produced, whereas incineration of fossil plastics emits CO_2_ that had been sequestered for millions of years” [66].

### 6.5. Materials

#### 6.5.1. Disposal of PHA

**Figure 15:**
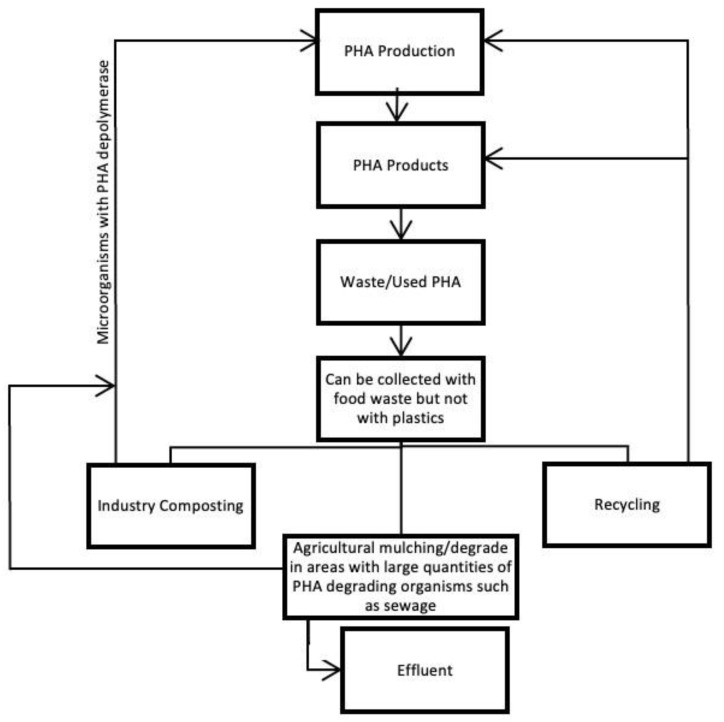
Waste management process of PHA. Adapted from “Bioplastics: A boon or bane?”, Renewable and Sustainable Energy Reviews, vol. 147. Elsevier Ltd., 1 September 2021 [31].

The types of microorganisms capable of degrading PHA are numerous, which makes this biopolymer easily compostable under industrial and nonindustrial conditions. Home composting is possible in this case, since the degradation of the polymer may start at 30 °C. PHA is known to improve the soil in which it decomposes, since it increases the diversity of microbes present in the soil; in turn, this also increases the composting efficiency, as well as the variety of materials able to be degraded. This means that the separation process of these materials does not need to be so stringent, saving time and money. The waste management process of this material is summarized in Figure 15. Industrial decomposition of PHA takes 124 ± 83 days; under anaerobic digestion, it takes about 31 ± 20 days, while improperly abandoning this type of material on the soil takes 1–2 years.

PHA is also degradable in aerobic lagoon water treatment systems, which house a wide variety of microorganisms. This process is even more efficient than the one occurring on the soil. It is also possible to degrade these materials in landfills, although a lot slower and not ideal [31,67].

Although it is possible, it is not feasible to recycle PHA since its properties at high temperatures are very unstable and, if not isolated from other materials, it might contaminate the final polymer. Chemical recycling is still a method under study; some materials such as herbicides and plasticizers, among others, can be made from products of the chemical recycling of PHA [31].

#### 6.5.2. Disposal of PLA

**Figure 16:**
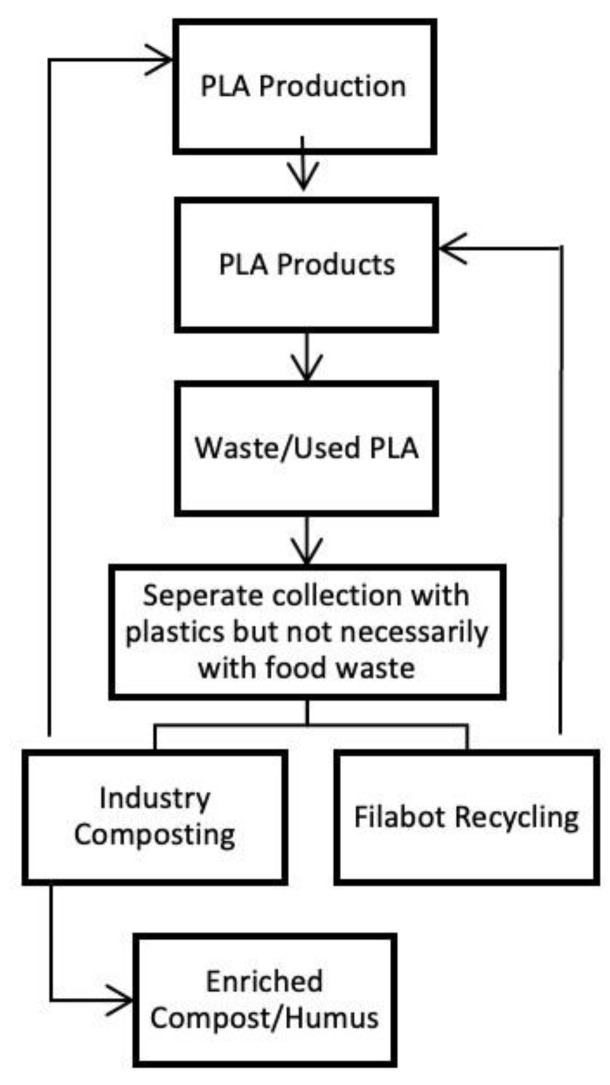
Waste management process of PLA. Adapted from “Bioplastics: A boon or bane?”, Renewable and Sustainable Energy Reviews, vol. 147. Elsevier Ltd., 1 September 2021 [31].

Initially, the objective of using PLA on single-use plastic items was to transit from petrochemical plastics to biodegradable bioplastics. However, currently, the disposal process of these materials is not any different from the conventional plastics, which is not the most effective method to use. Some manufacturers, such as NatureWorks, have clearly stated that the materials they produce PLA are to be industrially composted and have even explained how the process works. PLA is commonly used as a 3D printing filament, and there are several machines that convert used filament into a new one, making it possible to recycle these materials more efficiently. The waste management process of this material is summarized in Figure 16 [31].

It is hard to determine if a material is truly biodegradable; for example, in the case of PLA, the enzymes needed to degrade this biopolymer are not present in the natural environment. This means that to biodegrade PLA efficiently, the environment in which this process occurs must be controlled, especially its temperature and moisture. PLA needs to be at least 60 °C and with a lot of moisture to catalyse self-hydrolysis of the material. It is hard to achieve these conditions in home composting, which might lead to the production of inert materials. The conversion of the polymer to lactic acid is aided by the temperature and moisture available; this element is then used as a source of nutrients by several types of microorganisms. Under controlled industrial composting conditions, it takes 84 ± 47 days for the complete degradation of PLA; using anaerobic digestion takes about 423 ± 76 and 116 ± 48 days in mesophilic and thermophilic conditions, respectively, while in soil, 4–5 years are necessary for PLA to disappear.

The end product of the process should be a mix of humus, water and CO_2_. If not processed, the remaining lactic acid reduces the soil’s pH, affecting the development of microorganisms and their activity [31].

## 7. Degradation Process

This process may be anaerobic or aerobic, and according to the type of product obtained from the process, biomass or gas and minerals, it can be called complete biodegradation or mineralization, respectively. Both processes are influenced by endogenous (such as molecular weight, crystallinity and flexibility of the molecule) and exogenous (temperature, humidity, pH, availability of oxygen and enzymatic activity) factors, which may directly affect the entire process. The best outcome regarding the degradation of bioplastics is composting, since the final products of the process are a soil-like substance called humus, CO_2_, water and inorganic compounds, leaving no toxic residues (if no additives were used). However, it is important to consider that this is only achievable if the right conditions are met: precisely controlled temperature, humidity, oxygen, etc. Studies show that composting PLA under natural conditions equals approximately 10–20% efficiency when compared to the process under a controlled environment [68].

**Figure 17:**
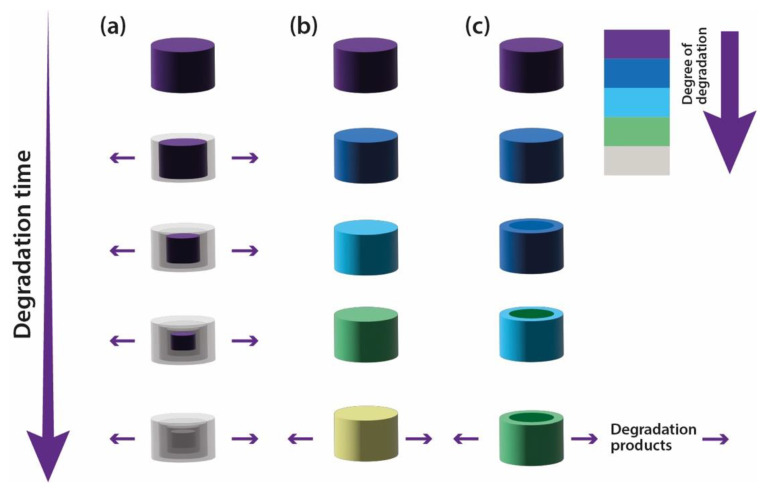
Degradation modes for degradable polymers: surface erosion (**a**), bulk degradation (**b**) and bulk degradation with autocatalysis (**c**). Adapted from [46].

Various degradation mechanisms degrade PHA and PLA; those include physical, chemical, oxidative, hydrolytic, enzymatic, microbial, photodegradation and thermodegradation mechanisms and have been studied by many researchers [69]. The different mechanisms in which the polymer degradation may occur are linked to the type of diffusion-reaction phenomenon taking place. When the absorption of water into the polymer is slower than the hydrolytic chain scission and the diffusion of the monomers into the surroundings of the plastic, it is said that the degradation is called surface erosion. As represented in Figure 17a, over time, a thinning of the plastic piece occurs without decreasing the internal molecular weight of the polymer.

Bulk degradation, on the other hand, happens when water penetrates the entire volume of the polymer, resulting in its molecular weight decrease throughout the piece matrix (Figure 17b). If this process does not occur in equilibrium, the erosion does not occur gradually (water does not penetrate the polymer to hydrolyse the chains, making it impossible for the monomers to diffuse out). In that case, it is possible that internal autocatalysis could take place via the carboxyl and hydroxyl end group by-products. The internal concentration of this autocatalysis may create an acidic gradient, resulting in a nucleus with a faster degradation rate than the polymer’s surface. The process evolves with the outer layer with a higher molecular weight than the piece’s interior, as shown in Figure 17c. Thus, this polymer is denominated as having a bimodal molecular weight distribution. In time, with the continuous diffusion of the inner polymer through the outer layer, the piece turns into a hollowed-out structure [46].

There is, however, an important notion to consider when referring to the degradation of plastics, which is the possibility of mixing additives with conventional plastics (such as PE, PP (polypropylene), PS, PET or PVC (polyvinylchloride)) to mimic the biodegradation process. These materials are called “oxo-biodegradable” or “oxo-degradable” plastics, and the additives used are usually transition metals such as nickel, iron, manganese and cobalt. Their main function is to make it easier for the polymer to break down into smaller pieces. The idea is to allow microorganisms to process the material and convert it into CO_2_ and the biomass. This degradation process is called oxo-degradation and is divided into two different stages: the first is related to the fragmentation of the polymer itself, which is an abiotic process where the prooxidant actions create an oxidative degradation of the polymer; in the second stage occurs in the biotic process where microorganisms convert the products of the previous stage into CO_2_ and the biomass. However, this process is hard to predict concerning the time frame in which it takes place, since it depends on the climate factors such as temperature and intensity of solar radiation.

Oxo-degradable materials, however, are not considered as compostable or recyclable, as defined according to the standards accepted by the industry (ASTM D6400—Standard Specification for Labelling of Plastics Designed to be Aerobically Composted in Municipal or Industrial Facilities; ASTM D6868—Standard Specification for Labelling of End Items that Incorporate Plastics and Polymers as Coatings or Additives with Paper and Other Substrates Designed to be Aerobically Composted in Municipal or Industrial Facilities; EN 13432—Requirements for packaging recoverable through composting and biodegradation—Test scheme and evaluation criteria for the final acceptance of packaging; ISO 17088—Specifications for compostable plastics; ASTM D5338—Standard Test Method for Determining Aerobic Biodegradation of Plastic Materials Under Controlled Composting Conditions, Incorporating Thermophilic Temperatures and ASTM D5929—Standard Test Method for Determining Biodegradability of Materials Exposed to Source-Separated Organic Municipal Solid Waste Mesophilic Composting Conditions by Respirometry); after the first stage of the process, the fragmented materials, although invisible, are still present in the environment as microplastics, and there is no guarantee that the entirety of the material will biodegrade or how long it takes.

In 2019, the European Parliament banned the use of oxo-degradable plastics. Until then, companies such as Pizza Hut, Nescafe, KFC, Tiger Brands, Tesco Barclay and Walmart usually used oxo-degradable materials in their products. On the other hand, countries such as the United States of Emirates, Saudi Arabia, Bahrain and Jordan regularly use oxo-biodegradable plastics. In Saudi Arabia, since April 2017, many single-use plastics have been made of these types of materials, and the intention is to expand this utilization. The available literature is divided regarding the eco-friendliness of these materials, and the companies that produce these polymers strongly defend their biodegradability [32,70,71,72].

## 8. Environmental Viability Assessment

Although bioplastics are known as a green alternative to conventional polymers, some drawbacks are making it a questionable choice. There are some bioplastics that only break down in specific conditions or when treated in municipal composters or digesters. When decomposed in composts, they release methane and CO_2_ into the atmosphere.

**Figure 18:**
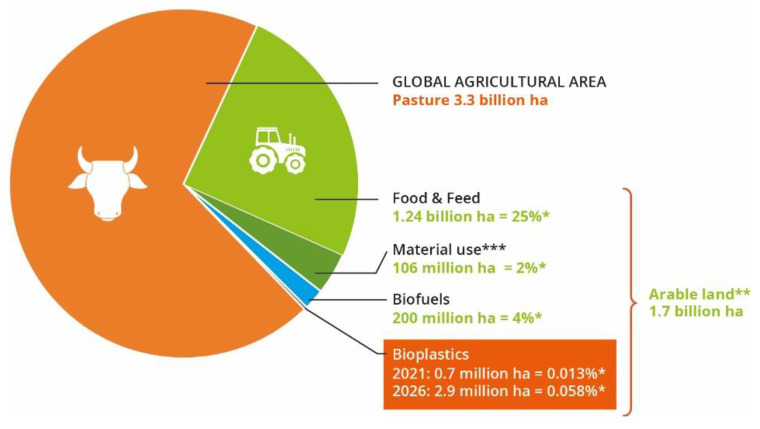
Land use estimation for bioplastics 2021 and 2026. Adapted from European Bioplastics, “BIOPLASTICS MARKET DEVELOPMENT UPDATE 2021”, * in relation to global agricultural area, ** including approx.. 1% fallow land, *** land-use for bioplastics is part of the 2% material use. https://docs.european-bioplastics.org/publications/market_data/Report_Bioplastics_Market_Data_2021_short_version.pdf (accessed on 29 December 2022) [6].

The production of bioplastics is also controversial, because some of them are made from plants which production requires the occupation of land that could be used to plant food. Statistics revealed that 0.7 million ha of agricultural land are used to produce bioplastics (Figure 18). This situation has consequences on food prices and in the economy of some countries [6,39].

Recently, some studies revealed that, when comparing the production of traditional plastics and bioplastics, the bioplastics production created more pollutants due to the use of pesticides and fertilizers when growing the crops, and bioplastics contribute more to ozone depletion than conventional plastics.

However, recent studies reveal that not only the production of PLA saves two-thirds of energy when compared to traditional plastics production, but also, the disintegration of this material does not increase CO_2_ in the atmosphere. This is possible, because the plant used as raw material to produce PLA absorbed the same quantity of CO_2_ as the quantity released during the degradation of this polymer. Plus, the degradation of PLA in landfills emits 70% less greenhouse effect gases than conventional plastics. The use of renewable energies also helps to make the final products greener.

The problem regarding using food sources as raw materials to produce bioplastics is easily avoided by using crop residues as the base production material, such as stems, straws, husks and leaves. By using alternative carbohydrate sources, this problem is averted. It is also possible to use kitchen waste, fish meal wastes and paper sludge as a source of carbohydrates to produce PLA, making it possible to help the waste management in big cities.

To correctly evaluate the environmental impact and viability of bioplastics, it is necessary to assess all the processes from initial production to the final disposal. An LCA (life cycle assessment) is usually used to do this. A cradle-to-grave analysis, for example, helps determine the impact of the use of certain materials and compare them with other ones, making it possible to evaluate the whole life of products from beginning to end and in each stage of utilization. Several scenarios are studied, for example, if it is better to recycle or compost the material, to determine the best possible solution. Recent studies revealed that incineration or landfilling of bioplastics is not a useful option. It was also concluded that using PLA and TPS reduces greenhouse emissions by 50 to 70%. PTT (polytrimethylene terephthalate) and bio-urethanes release 36 and 44% less greenhouse gases, respectively. Studies have shown that the issues observed during the production of bioplastics are still less harmful than the use of conventional plastics, and it is possible to address them verified during the production of bioplastics [2,31,39,73].

There are several factors important to consider when performing an LCA; environmentally, some of the most relevant ones are:

### 8.1. Abiotic Depletion

Minerals and fossil fuels are some of the system’s inputs that are important to consider, since the extraction of these materials affects the health of humans and the environment. For each extraction of these materials, the abiotic depletion factor is determined. This factor is measured based on kg of antimony (Sb) equivalents per kg of extracted mineral.

### 8.2. Global Warming

This is related to the amount of greenhouse gas emissions. Global warming is a hazard that severely affects the ecosystem, human health and material welfare. The absorption of infrared radiation changes the climatic patterns and increases the global average temperatures. This factor is measured based on its kg of CO_2_ equivalents per kg of emission.

### 8.3. Human Toxicity

This category does not include health risks in the work environment; the main concerns are related to toxic substances’ effects on the human environment. The purpose is to measure the human toxicity potentials, which may include the fate, exposure and effects of toxic substances for an infinite time. This factor is measured based on its kg of 1,4-DB (1,4-Dichlorobenzene) equivalents per kg of emission.

### 8.4. Freshwater Aquatic Ecotoxicity

Similar to human toxicity, the goal is to determine the fate, exposure and effects of toxic substances in the air, water and soil on fresh water. This factor is measured based on its kg of 1,4-DB equivalents per kg of emission.

### 8.5. Marine Aquatic Ecotoxicology

The logic used to determine this factor is the same as the human toxicity and the freshwater aquatic ecotoxicity; the characterization factor is the potential of marine aquatic toxicity of each substance emitted into the air, water or/and soil. This factor is measured based on its kg of 1,4-DB equivalents per kg of emission.

### 8.6. Terrestrial Ecotoxicity

Once again, the characterization factor is the potential of terrestrial toxicity of each substance emitted into the air, water or/and soil. This factor is measured based on its kg of 1,4-DB equivalents per kg of emission.

### 8.7. Photochemical Oxidation

Reactions between NOx (nitrogen oxides) and VOCs (volatile organic compounds) when in contact with UV (ultraviolet) light create photochemical oxidant smog, leading to the formation of ozone in the troposphere. This phenomenon depends on the metrological conditions and the background concentrations of pollutants. This factor is measured based on its kg of C_2_H_4_ (Ethylene) equivalents per kg of emission.

### 8.8. Acidification

Refers to the increase of potentially toxic elements or the decrease of pH by the deposition of pollutants such as SO_2_ (sulphur dioxide), NOx, HCl (hydrochloric acid), CO_2_ and NH_3_ (ammonia), which may affect soil, groundwater, surface water, organisms, ecosystems and materials. This factor is measured based on its kg of SO_2_ equivalents per kg of emission.

### 8.9. Eutrophication

The deposition of excessive nutrients in a soil or water system, especially phosphates and nitrates, usually leads to excessive algae growth, potentially damaging life forms in the system by affecting the ecosystem equilibrium. This factor is measured based on its kg of PO_4_^3−^ (Phosphate) equivalents per kg of emission [68,74,75].

## 9. Statistics of Bioplastics

**Figure 19:**
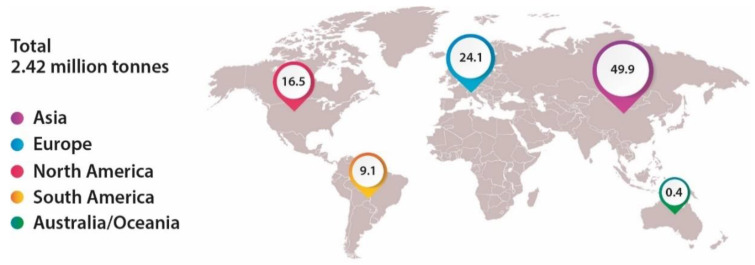
Global production capacities of bioplastics in 2021 (world map). Adapted from “Global production capacities of bioplastics in 2021 (world map)”, https://www.european-bioplastics.org/wp-content/uploads/2021/11/Global_Prod_Capacity2021_map.jpg (accessed on 29 December 2022) [76].

According to European Bioplastics, the production of bioplastics represents only 1% (2.42 Mt) of all plastic production, and the vast majority was produced in Asia, about 50%. Europe is the second continent with a greater capacity to produce bioplastics, as shown in Figure 19. The growth of the last few years is greatly influenced by the incentives of the European Commission to decrease the dependency on fossil fuels and transition to a circular economy [33,75].

**Figure 20:**
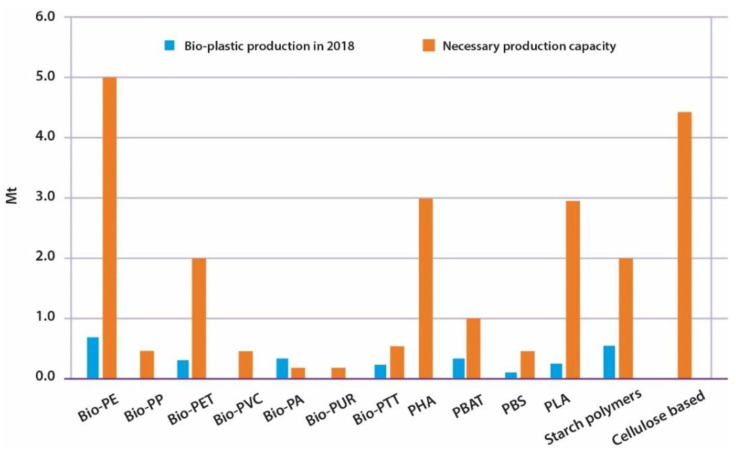
Current Bioplastic Packaging Production versus Necessary Production Capacity Source. Adapted from “The Unintended Side Effects of Bioplastics: Carbon, Land, and Water Footprints”, One Earth, vol. 3, no. 1. Cell Press, pp. 45–53, 24 July 2020 [77].

However, this growth is not yet sufficient to consider it possible to replace the use of petrochemical plastics with biopolymers. A study developed by Janis Brizga et al. (2020) revealed that, to achieve this on packaging application alone, it would be necessary to increase by 8.4 times some of the bioplastics production (Figure 20). Other bioplastics would even need to increase production by 100 times. Although these values are only theoretical and no economic feasibility and resource availability are considered, it is possible to observe that the production of bioplastics is still very far from what it would be necessary to replace petrochemical polymers [77].

Another study by F. Klein, A. Emberger-Klein, K. Menrad et al. (2019) evaluated the possible motives influencing the intention to purchase bioplastic products on the German market. The study concluded that green consumer values, attitudes towards bioplastic, product experience and interest in information are crucial factors influencing the community to buy bioplastic products. This means that information and communication are key aspects for people to choose to use bioplastics. To reinforce this, it was mentioned that “the purchase intention for bioplastic products measured for all German citizens is moderate at about 56%. In contrast, about 95% of the consumers with product experience intend to buy bioplastic products”. This means that, by increasing the promotion of bioplastics, the community becomes more aware of the importance of using these materials. One good starting point would be to set standards regarding the end-of-life usage of bioplastics, eliminating the confusion of some companies and consumers. Therefore, it is useful to consider the LCA analysis to support the choices made [78].

Numerous analyses can be done regarding the use of bioplastics and the consequences it has on important issues, such as fossil fuel consumption, economics, pollution, energy consumption and health.

### 9.1. Fossil Fuel Consumption

Using bioplastics instead of petrochemical ones can reduce fossil fuel consumption, since its production does not depend on them. Although it is not yet possible if all the petrochemical polymers were replaced by bioplastics and considering the energy used for its production as renewable, the consumption of fossil fuels would decrease 4% (3.49 million barrels a day). This value surpasses the daily consumption of every country except the United States, China and Japan. This simplified case reveals that the savings in oil consumption would be significant [79].

### 9.2. Economics

Generally, bioplastics are more expensive to produce than petrochemical polymers, but with the development of production techniques and the instability of oil prices, this reality tends to shift. For example, Mirel bioplastic made by Metabolix is about double the price of a petrochemical equivalent. The potential price stability is another benefit bioplastics have, and with the industry’s growth, the production prices of bioplastics should decrease [79]. To put it in perspective, Table 4 contains a list of typical market prices for common bioplastics inputs and polymers.

### 9.3. Energy Consumption

Several examples prove that the energy required to produce petrochemical polymers is higher than bioplastics. The total life cycle of HDPE requires 73.7 MJ kg^−1^ of plastic produced, LDPE uses 81.8 MJ kg^−1^ and PP 85.9 MJ kg^−1^. On the other hand, PHB requires 44.7 MJ kg^−1^ of plastic produced, PLA uses 54.1 MJ kg^−1^ and TPS 25.4 MJ kg^−1^. This represents significant consumption savings, and these values might have even greater differences due to the development of the techniques used to produce bioplastics. PLA energy requirement might reach values as low as 7.4 MJ kg^−1^.

Hypothetically, if all the PP used in the United States were replaced by PHB, PLA or TPS, the annual energy savings would be 363, 280 and 529 PJ, respectively. Considering that it is necessary to use 31,250 metric t of coal to produce 1PJ of energy, the savings would be enormous [79].

### 9.4. Pollution

Less pollution is produced by the production of bioplastics when compared with petrochemical polymers, not only by requiring less energy to be produced and emitting less CO_2_ but also by the possibility of recycling these materials. Bioplastics need to have their own recycling process, but they are not yet produced in enough quantities for that to happen; for this reason, they are considered a contaminant to the process. In perspective, if 0.1% of bioplastic material mixes with PET during its recycling process, the entire batch would become useless. It is important, however, to notice that bioplastics are as easily recyclable as petrochemical ones; they just need to be processed separately [79].

### 9.5. Health

So far, bioplastics have not been linked to any type of health problem. However, it is important to consider that the bioplastic industry is still under development, which means that further studies could reveal some issues with the use of these materials. It is essential to consider the use of pesticides, used during crop growth, and plasticizers. The problem with plasticizers is better understood than the bioplastics themselves. Nevertheless, bioplasticizers with low toxicity can be used in bioplastics [79].

## 10. Advantages and Disadvantages

Petrochemical-based polymers are a type of material in the process of extinction for several motives, such as the environmental hazard they usually provoke; their waste management difficulties; the risk of toxicity for other materials, animals and plants; the limited oil and gas resources and their increasing prices. However, these disadvantages are balanced by the low cost and high-speed production of pieces made of these materials, their high mechanical performance, good barrier properties and good heat stability.

Based on those disadvantages, the utilization of bioplastics works as an alternative. Bioplastics have a much lower carbon footprint, although if the polymer is biodegradable, the CO_2_ stored during the formation of the raw material will be released when it degrades, unlike the permanent bioplastics, which can be recycled many times while still storing the CO_2_ absorbed, but this released CO_2_ is compensated for by the fact that, during the growth of some types of bioplastics raw materials (plants), they absorbed the same quantity of this gas from the atmosphere. For example, 1 kg of bioplastic resign produces about 0.49 kg of CO_2_, while petrochemical-based polymers produce 2 to 3 kg of CO_2_. They make it possible for every country to produce polymeric materials without depending on countries with petroleum reserves. The production of bioplastics also takes less energy than conventional plastics.

However, the overall costs to produce bioplastics are higher than the petroleum-based polymers; nevertheless, it is important to consider the possibility of implementing several cost reduction mechanisms. Currently, bioplastics are considered to contaminate the recycling process of other plastics, but this problem is easily overcome by separating it from conventional plastic from the beginning of the process. The production of bioplastics might also reduce the available resource reserves usually used as food by-products. The composting possibility of some bioplastics might create some confusion. These materials are not compostable in the same way as conventional food waste, as it might be interpreted. The process requires controlled conditions that can only be achieved under an industrial composting site. The production, usage and waste management of bioplastics are not yet under any specific legislation in some countries [2,4,33,80].

## 11. Regulation

**Figure 21:**
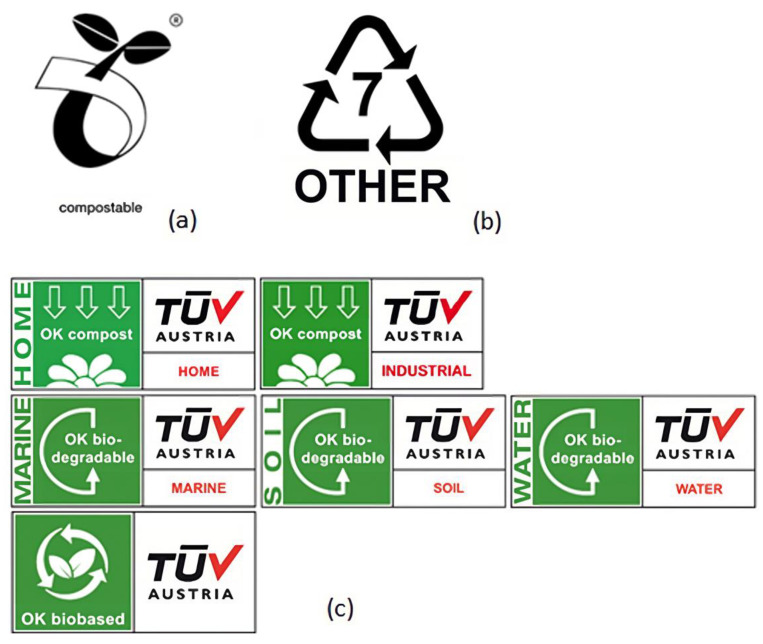
Labels currently used as compost, biobased and biodegradable polymers by European Bioplastics (**a**), ASTM International (**b**) and the Vinçotte/TÜV AUSTRIA Group (**c**) [32,73,81].

Currently, bioplastics and biodegradable plastics are identified by the ASTM International Resin Identification Coding System as part of group 7 or ‘other’ (Figure 21b), an identification system developed by the Society of the Plastics Industry in 1988 and administered by ASTM International since 2008. This means that the polymers included in this category do not have specified characteristics, and their management process is not defined. A solution to this problem would be to globalize a symbol easily identifiable identifying polymers classified as compostable or biodegradable according to proper standards, such as ASTM D6400, ASTM D6868, EN 13432, ISO 17088, ASTM D5338 and ASTM D5929. For example, the European Bioplastics created the Seeding logo (Figure 21a) as a label to identify compostable polymers according to the EN 13432 standard. Usually, this label goes along with one other created by Vinçotte (taken over by TÜV AUSTRIA Group), the “OK compost home“, “OK compost industrial“, “OK marine biodegradable“, “OK soil biodegradable“, “OK water biodegradable“ and “OK biobased“ (Figure 21c), because these should guarantee complete biodegradability in the light of specific requirements [31,39,73].

**Figure 22:**
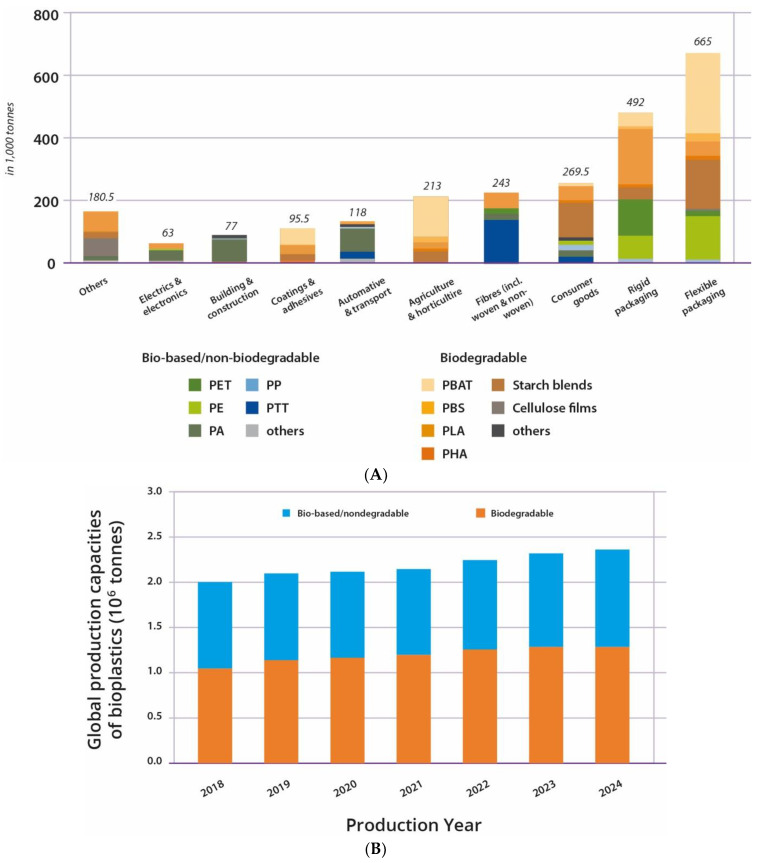
Global production capacities of bioplastics 2021 (by market segment) (**A**) and growth of bioplastic production in recent years (**B**). Adapted from European Bioplastics, “Bioplastics market data”, https://www.european-bioplastics.org/market/ (accessed on 29 December 2022) [82].

Some companies already use bioplastics in their products; for example, Coca-Cola uses bioplastics in its packaging. Recently, Coca-Cola revealed that they are launching their first 100% biobased bottle (excluding the cap and label), made of bio-PX (plant-based paraxylene) converted to bio-PTA (plant-based terephthalic acid) and using a new process, commercially viable developed by Virent. PTA is one of the main components of PET (70%); the remaining 30% is MEG (monoethylene glycol), which was already being produced from sugarcane. Since then, Coca-Cola has allowed non-competitive companies to use the technology and brand in their products, from Heinz Ketchup to the fabric interior in Ford Fusion hybrid cars. This, however, is done by their own initiative. To incentive the implementation of bioplastics as a substitute for conventional plastics, it is important to encourage companies with certain promotion and support mechanisms, such as regulations and monetary inducement. Financial profit is one of the main reasons a company invests in something. However, this principle is not yet applicable to the industry of bioplastic production. This is due to the fact that consumers are not expected to buy bioplastic goods more expensive than conventional plastics just because they might be more eco-friendly. Cereplast, a TPS producer, estimated that when the oil price reaches around 95 USD per barrel, the company’s production costs will be lower than petrochemical plastics, and the demand for bioplastics will increase. According to European Bioplastics, the packaging is still the main application of bioplastics, 48% (1.15 million t) of the total bioplastics market in 2021 (Figure 22) [32,82].

With the objective of creating a simple tool for companies to select the best possible solution when selecting a good bioplastic material for food packaging, the Association of Organic Food Producers in Germany, created an Internet tool called the “Biokunststoff-Tool”. This program evaluates areas such as ecology, social acceptability, safety, quality and technology of several bioplastics, which makes it possible to select environmentally and socially responsible production methods and materials.

To make the transition to bioplastics easier, some legal changes should be applied. For example, create a strict separation between biobased and biodegradable plastics using marks and labels as in the previous examples. This facilitates the implementation of waste treatment processes specialized in these materials. However, before that, the labelling information should be spread through consumers and waste management companies [32,83].

## 12. Conclusions

Bioplastics are materials with great potential for development. Although it is not yet used industrially on a large scale, the ecological advantages of using this material compared with other plastics are enormous. Less chemical pollution, less energy consumption and less CO_2_ emissions are some of the major drivers of a transition to a circular economy using bioplastics. On the other hand, its production is costly, and they do not have a proper recycling process. The limitations derived from the mechanical characteristics may be overcome and adapted to the intended application using additives. The disadvantages will be gradually overcome with the development of new technology and further research, over time, regarding these materials. To speed up the process, it is essential to spread the information about bioplastics to companies and consumers to convince the consumers that these materials are an excellent alternative to petrochemical-based polymers. In fact, global production capacities have been increasing and show strong growth trends; as such, new applications can be foreseen when large amounts of biopolymers are available for large-scale productions. However, biopolymers and bioplastics are not exempt from sustainability issues. Recycling these materials is a controversial debate because of their biodegradability-specific conditions, their potential methane emissions with a negative climate impact when discarded in landfills and potential contamination of the petroleum-based recycling stream. Other issues are related to land use due to ethical reasons about the potential competition with food resources. These and other concerns are all essential aspects to debate, at the academic, civil, economic and political levels. Laws, norms and regulations related to the environment have the potential to reduce the impacts on the environment. The LCA analysis can be used as a universal tool to assess those impacts and to support political decisions, therefore paving the way for a green transition.

## Figures and Tables

**Table 1:** Comparison of typical biodegradable polymer physical properties with LDPE, PS and PET. [2,17,18,19,20,21,22,23,24,25,26].

	Tg (°C)	Tm (°C)	MFR (g/10 min)	Tensile Strength (MPa)	Tensile Modulus (MPa)	Elongation Break (%)	Flexural Strength (MPa)	Flexural Modulus (GPa)	Izod Impact Strength (J/cm)
LDPE	−100	98 to 115	0.25 to 2300	8 to 20	300 to 500	100 to 1000	9.03 to 932	0.0248 to 1.45	0.343 to 5340
PCL	−60	59 to 64	-	4 to 28	390 to 470	700 to 1000	-	-	-
Starch	-	110 to115	1.98	35 to 80	600 to 850	580 to 820	-	-	-
PBAT	−30	110 to 115	-	34 to 40	-	500 to 800	-	-	-
PTMAT	−30	108 to 110	-	22	100	700	-	-	-
PS	70 to 115	100	1.2 to 100	34 to 50	2300 to 3300	1.2 to 2.5	28 to 106	0.894 to 3.60	0.107 to 2.14
Cellulose	-	-	-	55 to 120	3000 to 5000	18 to 55	-	-	-
PLA	40 to 70	130 to 180	0.20 to 92.8	48 to 53	3500	30 to 240	0.170 to 159	0.167 to 13.8	0.105 to 8.54
PHB	0	140 to 180	17 to 20	25 to 40	3500	5 to 8	18	16	
PHA	−30 to 10	70 to 170	-	18 to 24	700 to 1800	3 to 25	40	2	0.260
PHB—PHV	0 to 30	100 to 190	-	25 to 30	600 to 1000	7 to 15	-	-	1
PVA	58 to 85	180 to 230	17 to 21	28 to 46	380 to 530	-	-	-	-
Cellulose Acetate	-	115	-	10	460	13 to 15	27 to 72	0.08 to 2.62	0.480 to 4.50
PET	73 to 80	245 to 265	3.5 to 65	48 to 72	200 to 4100	30 to 300	55.3 to 135	0.138 to 3.50	0.139 to 100
PGA	35 to 40	225 to 230	-	890	7000 to 8400	30	-	-	-
PEA	−20	125 to 190	-	25	180 to 220	400	-	-	-

**Table 2:** Major bioplastic classes, some properties and average degradation time in different environments [27,28,29]. (**√/X:** present/absent).

Bioplastic	Manufacturer	Properties		Applications	Degradable		Degradation Time (Days)
Starch—TPS	Novamont (Italy) Livan (Canada) Ever Corn (Japan) Plaststa rch (USA)	**√**	Thermoplastic	Packaging; Food trays; Trash bags; Flowerpots	**√**	In Water	72–236
		**√**	Gas Barrier				
		**X**	UV-Resistant				
		**√**	Biocompatible		**√**	In Soil	
		**X**	Thermostable				
		**√**	Elastic		**√**	Industrial Compost	
		**√**	Rigid				
		**X**	Hydrophobic				
Polyhydroxyalkanoates—PHA, PHB and PHV	Minerv (Italy) Biogreen (Japan) Biocycle (Brazil) Green Bio (China)	**√**	Thermoplastic	Packaging; Adhesives; Fibers; Medical implants	**√**	In Water	15–280
		**√**	Gas Barrier				
		**√**	UV-Resistant				
		**√**	Biocompatible		**√**	In Soil	
		**X**	Thermostable				
		**√**	Elastic		**√**	Industrial Compost	
		**√**	Rigid				
		**√**	Hydrophobic				
Polylactide—PLA	Nature Works (USA) Biofoam (The Netherlands) Ingeo (USA) Hisun (China) Biofront (Japan)	**√**	Thermoplastic	Packaging; Textiles; Medical implants; Films	**X**	In Water	28–98
		**√**	Gas Barrier				
		**√**	UV-Resistant				
		**√**	Biocompatible		**√**	In Soil	
		**X**	Thermostable				
		**√**	Elastic		**√**	Industrial Compost	
		**√**	Rigid				
		**√**	Hydrophobic				
Cellulose-Based Polymers	Natural flex (UK) Tenite (USA) Biograde (Germany) Sateri (China)	**X**	Thermoplastic	Wound dress; Textiles; Air filters; Coatings	**√**	In Water	14–154
		**X**	Gas Barrier				
		**X**	UV-Resistant				
		**√**	Biocompatible		**√**	In Soil	
		**√**	Thermostable				
		**X**	Elastic		**√**	Industrial Compost	
		**√**	Rigid				
		**X**	Hydrophobic				
Protein-Based Polymers		**√**	Thermoplastic	Cast film; Injection moulding; Extrusion sheets; Compression moulding	**√**	In Water	36–50
		**X**	Gas Barrier				
		**√**	UV-Resistant				
		**√**	Biocompatible		**√**	In Soil	
		**√**	Thermostable				
		**√**	Elastic		**√**	Industrial Compost	
		**√**	Rigid				
		**√**	Hydrophobic				

**Table 3:** Processing possibilities of typical commercial biopolymers. Adapted from “Poly-Lactic Acid: Production, applications, nanocomposites, and release studies”, Comprehensive Reviews in Food Science and Food Safety, vol. 9, no. 5, pp. 552–571, September 2010 [2].

	Injection Moulding	Extrusion	Extrusion Blow Moulding	Cast Film Extrusion	Blow Moulding	Fibre Spinning	Thermo-Forming
Starch	x	x	x	x			
Cellulose	x	x			x		
PHB	x	x	x	x	x		x
PHB-PHV	x	x	x	x	x	x	x
PLA	x	x		x	x	x	x
PBS	x	x					
PCL	x	x	x		x	x	x
PBST	x	x		x			x
PBAT		x	x	x			
PTMAT		x	x	x		x	
PVA	x		x	x		x	x
PP, PE + additives	x	x		x	x	x	x
Starch + PVA	x	x		x	x	x	
Starch + cellulose acetate	x	x	x		x		x

**Table 4:** Bioplastics and raw material prices (2018). Adapted from “Green Bioplastics as Part of a Circular Bioeconomy”, Trends in Plant Science, vol. 24, no. 3. Elsevier Ltd., pp. 237–249, 1 March 2019 [23].

Bioplastic	Approximate Price (EUR/kg)
Corn starch	0.34
Lactic acid	1.14
Unbleached dissolving pulp	1.26
Soybean protein isolate	2.02
PLA	1.72
PHA	2.49
Cellulose acetate	21.50

## Nomenclature

1,4-DB	1,4-Dichlorobenzene	3DP	3D printing
Bio-PETG	Bio Polyethylene Terephthalate Glycol	Bio-PTA	Plant-based Terephthalic Acid
Bio-PX	Plant-based Paraxylene	CNC	Cellulose Nano Crystals
C_2_H_4_	Ethylene	CO_2_	Carbone Dioxide
CPE	Chlorinated Polyethylene	FDM	Fused Deposition Modeling
H_2_O	Water	HCl	Hydrochloric Acid
HDPE	High-Density Polyethylene	LCA	Life Cycle Assessment
LDPE	Low-Density Polyethylene	MEG	Monoethylene Glycol
NFC	Nano Fiber Cellulose	NH_3_	Ammonia
NOx	Nitrogen Oxides	MSW	Municipal Solid Waste
PA	Polyamide	PBAT	Polybutylene Adipate Terephthalate
PBS	Polybutylene Succinate	PBST	Polybutylene Succinate
PBT	Polybutylene Terephthalate	PCL	Polycaprolactone
PE	Polyethylene	PEA	Polyethylene Adipate
PET	Polyethyleneterephthalate	PGA	Polyglycolic Acid
PHA	Polyhydroxyalkanoates	PHB	Polyhydroxy Butyrate
PHV	Polyhydroxybutyrate	PLA	Polylactide
PO_4_^3−^	Phosphate	PP	Polypropylene
PS	Polystyrene	PTMAT	Polytetramethylene Adipate Terephthalate
PTT	Polytrimethylene Terephthalate	PVA	Polyvinyl Alcohol
PVC	Polyvinylchloride	PVdC	Polyvinylidene Chloride
SO_2_	Sulfur Dioxide	SPI	Soy Protein Isolate
Tg	Transition Temperature	Tm	Melting Temperature
TPLA	Talc-Injected PLA	TPS	Thermoplastic Starch
UV	Ultraviolet	VOC	Volatile Organic Compound
